# Virtual reality as a training tool for athletes with intellectual disabilities: a study protocol

**DOI:** 10.3389/fspor.2026.1756489

**Published:** 2026-02-06

**Authors:** Ivelina Kirilova, Milena Kuleva, Stefka Djobova, Mariana Borukova

**Affiliations:** 1 Department of Adapted Physical Activity, National Sports Academy “Vassil Levski”, Sofia, Bulgaria; 2 Department of Information Technology for Motion Analysis, National Sports Academy “Vassil Levski”, Sofia, Bulgaria; 3 Department of Basketball, Volleyball, Handball, National Sports Academy “Vassil Levski”, Sofia, Bulgaria

**Keywords:** adapted sport, education, intellectual disabilities, Simulator Sickness Questionnaire (SSQ), virtual reality

## Abstract

**Introduction:**

Immersive virtual reality (VR) is increasingly used in education and rehabilitation, yet little is known about its feasibility and tolerability among athletes with intellectual disabilities (ID). Understanding emotional, behavioral, and physical responses is essential before integrating VR into adapted sport training.

**Methods:**

This prospective, observational case-series included six male athletes with mild to moderate ID participating in adapted basketball training. Each athlete completed three sessions of low-motion, non-interactive 360° basketball-themed VR using the Meta Quest 3 headset. Emotional responses, behavioral indicators, static and dynamic balance, SSQ-aligned observational symptoms, and detailed usability logs were collected before, during, and after VR exposure. Descriptive statistics were used to summarize patterns across sessions.

**Results:**

Emotional grading remained predominantly positive across all sessions, with no VR-related negative affect. Engagement increased while distraction and hesitation decreased over time, indicating improved familiarity with immersive environments. Balance performance remained stable post-VR, with no signs of postural deterioration. SSQ-aligned monitoring showed negligible symptoms (98.1% scored zero), and no nausea occurred. Usability challenges were minor and primarily related to controller use, video initiation, and battery life.

**Discussion:**

Short, structured VR exposure was well tolerated and operationally feasible for athletes with ID. Findings support VR's potential as a safe and engaging supplementary tool in adapted basketball training, warranting further research with larger and more diverse samples.

## 1 Introduction

Virtual reality (VR) technologies are becoming increasingly popular across education and rehabilitation, as well as in sports training, where immersive, interactive environments can provide engaging, repeatable, and low-risk learning experiences (1–3). Recent reviews highlight a rapid expansion of VR-based interventions for individuals with intellectual disabilities (ID), particularly since 2021, with most studies focusing on adults with mild to moderate ID and on practical or adaptive skill development (1).

Although these interventions commonly use high-immersion devices (e.g., head-mounted displays with 6-DoF tracking) and structured teaching formats (e.g., task-segmented, step-by-step VR training protocols), personalized applications and training approaches targeting social, conceptual, or sport-specific skills remain limited. Persistent challenges, including small sample sizes, methodological variability, and limited diversity, highlight the need for more inclusive research reflecting diverse ID profiles and for context-specific studies conducted in real-world settings such as sport.

As VR gains traction in healthcare and rehabilitation, interest has grown in its potential to support individuals with intellectual and developmental disabilities (4). Evidence suggests that VR can enhance physical, cognitive, and emotional skills (2), a finding particularly relevant for athletes with ID seeking to build independence, confidence, and performance capacity. However, successful implementation requires careful attention to usability, safety, and long-term practicality as technologies continue to evolve (5). Understanding how athletes with ID respond emotionally, behaviorally, and physiologically to immersive VR is, therefore, a critical prerequisite for its meaningful integration into sport environments.

Individuals with ID exhibit diverse cognitive, perceptual, and motor profiles that can influence their interaction with digital technologies. Differences in attention regulation, visuospatial processing, executive functioning, and sensory integration may affect how VR environments are perceived and tolerated (2). VR has been proposed as a valuable solution to challenges such as generalization and transferring learned skills to real-world contexts. By offering ecologically valid yet risk-free environments that can be repeated as needed, immersive VR has shown promise in supporting adaptive behavior, daily living skills, and cognitive functioning (1, 6–8). Fully immersive systems, in particular, can enhance attention, reduce distraction, and promote motivation through rich, engaging visual content (9, 10).

Despite these benefits, individuals with ID may demonstrate widely varying levels of tolerance to VR. Some users experience sensory overload, disorientation, or cyber sickness, making safety monitoring essential. Traditional self-report measures, such as the Simulator Sickness Questionnaire (SSQ), are frequently used to track VR-induced discomfort but may not be valid or accessible for athletes with ID, who often struggle to interpret or communicate internal states (11). Consequently, researchers recommend incorporating alternative methods, such as behavioral observation, simplified emotion rating tools, and objective physical indicators like balance performance (3), to accurately detect subtle signs of discomfort.

Although VR is increasingly used in cognitive, educational, and behavioral training for individuals with ID (1, 12–14), its application in sport contexts remains limited. Existing research addressing motor development or rehabilitation generally targets older adults or clinical populations rather than trained athletes (10). However, sport settings, particularly team sports such as basketball, offer rich opportunities for skill development, social participation, and physical confidence. Basketball requires spatial awareness, coordination, rapid decision-making, and scenario recognition, all of which could be supported through immersive, visually driven VR experiences. These sport-specific characteristics are especially relevant in adapted sports training, where practice conditions are intentionally modified through adjustments in rules, equipment, and instructional support to meet the participation needs of athletes with ID. VR may help athletes rehearse tactical patterns, develop familiarity with game situations, or practice perceptual-cognitive skills without physical risk.

Before integrating VR into adapted sports training, however, it is essential to evaluate whether athletes with ID can tolerate immersive environments without adverse emotional or physiological reactions. Prior research emphasizes the importance of monitoring psychological safety, as individuals with ID may experience anxiety or sensory sensitivity within unfamiliar environments (3). Studies generally report positive reactions to VR among individuals with mild to moderate ID, but emphasize the need for guided support and gradual familiarization (2).

Given the limited research on immersive VR in adapted sport, the present study uses Meta Quest 3 headset to deliver simplified 360° basketball-related content. Athletes' emotional, behavioral, and physical responses to immersive VR were documented alongside technical and usability challenges encountered during implementation. The study aims to support evidence-informed implementation of VR in adapted basketball training by providing feasibility-oriented data on emotional tolerance, behavioral adaptation, balance stability, and device usability in athletes with intellectual disabilities.

## 2 Methods

### 2.1 Study design

The study was designed as a small-scale prospective observational case study with a feasibility-oriented focus. This design was selected to document how athletes with intellectual disabilities respond emotionally, behaviorally, and physically to immersive VR exposure under real-world adapted basketball training conditions. An observational approach was considered appropriate given the exploratory nature of the research questions, which aimed to assess tolerability, usability, and implementation constraints rather than to test intervention efficacy (15–17).

Accordingly, the results are interpreted descriptively and are intended to characterize feasibility-related outcomes, including emotional acceptance, behavioral adaptation, balance stability, and practical usability of the VR system across repeated sessions. The study was not designed to evaluate training efficacy or performance effects, and no causal inferences are drawn from the findings.

Ten athletes were initially screened; following exclusions due to visual incompatibility, lack of parental consent, balance-related contraindications, or behavioral indications of reduced readiness, six participants met all inclusion criteria and completed the full VR protocol (Table 1).

**Table 1:** Participant characteristics.

Study ID	Age	Sex	ID level	Years of basketball	Other condition
Athlete 1	20	m	Moderate to severe	7	Disorder of intellectual development
Athlete 2	16	m	Mild	5	ADHD, Disorder of intellectual development
Athlete 3	16	m	Mild	2	ASD, Disorder of intellectual development
Athlete 4	16	m	Moderate to severe	6	ASD, Disorder of intellectual development
Athlete 5	23	m	Mild	7	Disorder of intellectual development
Athlete 6	23	m	Moderate	3	ASD, Disorder of intellectual development

Participants were recruited from a single adapted basketball program in which all athletes trained regularly together. The research team, who were also involved in coaching and daily training activities within the program, invited athletes to participate in the study. All athletes were familiar with one another and had an established training relationship prior to the study. Importantly, the athletes also had an existing relationship with the research team, resulting in a high level of familiarity and trust. This context facilitated participant engagement and supported smooth implementation of the VR protocol, including headset fitting and continuous monitoring procedures.

All participants were male, with a mean age of 19.0 years (SD = 3.5; range 16–23). ID levels included mild (*n* = 3), moderate (*n* = 1), and moderate-to-severe (*n* = 2). Athletes had an average of 5.0 years of basketball participation (SD = 2.0; range 2–7). Four participants presented comorbid neurodevelopmental conditions (ASD *n* = 3; ADHD *n* = 1). None of the participants was reported with epilepsy, seizures, frequent headaches, cardiovascular disease, or visual impairments. Two athletes had prior VR exposure; none reported as having a history of motion sickness.

This sample represented an athletically active, yet clinically heterogeneous, group suitable for examining VR tolerability and user experience in adapted sports training.

### 2.2 VR equipment and software

Immersive sessions were delivered using the Meta Quest 3 standalone headset with inside-out tracking, six degrees of freedom (6-DoF), and high-resolution pancake-lens displays. All sessions used non-interactive 360° basketball-related videos specifically created by the research team to ensure high ecological validity, stable optical flow, and minimal sudden movement.

The VR stimuli consisted of 360° video recordings depicting basketball-related environments relevant to adapted sport training. Videos were recorded in real basketball settings using a 360° camera positioned at standing eye level, providing a first-person visual perspective comparable to that of an athlete on the court. Scenes included static court views, slow movement through the playing area, and observational perspectives of basketball-specific contexts.

Visual realism was high in terms of fidelity and spatial representation, whereas motion dynamics were intentionally controlled to minimize abrupt accelerations, camera rotations, and visual transitions. Videos were edited to remove overlays, interactive elements, and rapid scene changes, prioritizing sensory stability over task complexity. This low-demand format was selected to reduce sensory overload and eliminate the need for controller interaction.

Content was streamed via Oculus casting to an external monitor, enabling real-time supervision, troubleshooting, and safety monitoring.

### 2.3 Procedure

Each athlete completed three VR sessions as part of their regular adapted basketball practice schedule. Sessions followed a fully standardized sequence consisting of pre-VR assessments, VR exposure, and post-VR assessments.

During VR exposure, athletes were not required to perform any specific virtual or real-world tasks. The VR experience was intentionally designed as a passive, observational exposure, in which athletes stood quietly and viewed non-interactive 360° basketball-related video content. No instructions related to performance, navigation, or task completion were provided. This approach was selected to capture spontaneous emotional, behavioral, and postural responses to immersive visual stimuli without introducing additional cognitive or motor demands.

Controllers were not required for the VR exposure and were intentionally excluded from the protocol to minimize interaction demands. However, in some sessions, controllers were physically present due to system default settings, leading to occasional accidental button activation or menu interruptions.

Screen prompts referred exclusively to system-generated interface messages (e.g., pause notifications, boundary alerts, or playback interruptions) and did not represent task-related instructions or user guidance.

All VR sessions were conducted in a quiet, dedicated indoor room adjacent to the regular training facility. The space was free of external distractions, provided sufficient open floor area for safe standing, and allowed stable headset tracking and casting to an external monitor. Lighting conditions were consistent across sessions and suitable for VR operation.

Athletes remained seated throughout VR exposure. Seating was selected to ensure postural stability and participant safety during immersive viewing, and to minimize unintended body movements that could interfere with headset tracking or induce discomfort.

The total duration of each session was approximately 25–30 min per athlete, comprising pre-session assessments (emotional rating and balance tasks; ∼5–7 min), headset fitting and calibration (∼3–5 min), VR exposure (∼7–10 min), and post-session assessments and debriefing (∼5 min).

#### 2.3.1 Pre-session preparation

Familiarization: Before the first session, athletes were introduced to the headset through a brief tactile and visual familiarization period. This ensured comfort with the device and reduced anxiety for VR-naive users.

Pre-VR Emotional Assessment: Athletes self-reported their emotional state using a three-option emoji scale (Good-green, Neutral-yellow, and Bad-red). This accessible format included visual symbols, color coded, and simplified verbal prompts.

Pre-VR Balance Assessment: Two balance tasks were administered and recorded:

Two balance tasks were administered and video recorded. Static and dynamic balance were evaluated using structured observational tasks scored within a feasibility-oriented framework developed for this adapted sports context.

Static balance was assessed during quiet standing with feet close together, arms extended laterally, and eyes open for approximately 20–30 s. Dynamic balance was assessed using a heel-to-toe walking task along a straight 2-meter line, performed at a self-selected pace. All trials were video recorded to allow detailed *post-hoc* analysis.

Balance performance was evaluated using a two-step scoring procedure. First, predefined observable indicators were rated and summed to generate raw balance scores. For static balance, indicators included sway amplitude, foot stability, arm compensation, and postural alignment, yielding a raw score range of 0–12, where lower scores indicated better stability (0–3 = excellent stability; 4–6 = adequate stability; 7–9 = unstable; 10–12 = poor stability or loss of control). These summed scores were then mapped onto a 0–3 ordinal scale, where 0 represented stable, well-controlled performance (raw score 0–3), 1 indicated minor instability with occasional postural corrections (4–6), 2 indicated moderate instability with frequent compensatory movements (7–9), and 3 indicated pronounced instability, such as marked sway, step deviations, or need for external support (10–12).

For dynamic balance, indicators included step symmetry, trunk control, direction-change control, foot placement accuracy, and gait flow/coordination, yielding a raw score range of 0–15, where lower scores reflected better coordination (0–4 = excellent coordination; 5–8 = adequate balance; 9–12 = reduced dynamic stability; 13–15 = unstable or increased risk of fall). These summed scores were likewise mapped onto the same 0–3 ordinal scale, with thresholds adjusted accordingly (0–4 = score 0; 5–8 = score 1; 9–12 = score 2; 13–15 = score 3).

This scoring framework was not intended as a validated clinical balance assessment but as a structured, feasibility-oriented method for detecting short-term postural changes before and after VR exposure in athletes with intellectual disabilities.

To ensure scoring consistency, at least two trained observers completed a calibration process prior to data collection, including joint review of example recordings and discussion of scoring anchors. During the study, 25% of recordings were independently double-scored, yielding 90% exact agreement across parallel ratings. Any discrepancies were resolved through consensus review, ensuring stable and reliable scoring within an applied sport setting.

Although raw scores were additionally mapped onto a 0–3 ordinal severity scale for descriptive classification, all analyses and reported results are based on summed raw balance scores to preserve sensitivity to change. Headset Fitting and Calibration: The headset was adjusted individually for interpupillary distance, strap tension, visual clarity, comfort, and stability.

Researchers verified clarity using simple Yes/No questions and observed facial cues. Casting ensured continuous monitoring of the athlete's field of view, enabling rapid correction of misalignment, tracking errors, or user confusion.

#### 2.3.2 VR exposure (7–10 min)

The duration of VR exposure was limited to 7–10 min per session to prioritize feasibility, tolerability, and participant safety during initial immersive exposure. Short exposure durations have been recommended in exploratory VR studies and feasibility research to reduce the risk of sensory overload, attentional fatigue, and VR-induced discomfort, particularly among users with limited prior VR experience or heightened sensory sensitivity (5, 11). Studies involving individuals with intellectual disabilities further emphasize the importance of brief, structured exposure and gradual familiarization to support emotional comfort and sustained engagement (1, 2). Given the feasibility-oriented nature of the present study, longer exposure durations were intentionally avoided to allow close monitoring of emotional, behavioral, and postural responses and to minimize cumulative effects across repeated sessions (15, 17).

Trained observers documented real-time behaviors across five domains: engagement, distraction episodes, hesitation events, verbal expressions, and physical cues.

The behavioral coding framework was adapted from established VR engagement literature and observational models used in adapted physical activity, ensuring that the selected categories reflect meaningful indicators of attention, comfort, and cognitive load in individuals with ID (2, 6, 8, 15, 17). Each domain was operationalized using predefined behavioral descriptors and frequency-based scoring anchors. Behavioral indicators were scored using frequency-based anchors defined *a priori* for each domain.

Engagement was scored based on sustained visual orientation toward the VR content and spontaneous interest behaviors (e.g., verbal comments, focused posture), with higher scores indicating longer and more consistent engagement. Distraction was coded as observable shifts of attention away from the VR content (e.g., looking away, interacting with the environment), with higher scores reflecting more frequent episodes. Hesitation was defined as pauses, uncertainty, or delayed responses during VR exposure. Verbal expressions included spontaneous comments, exclamations, or questions related to the VR content, and physical cues captured observable bodily reactions such as gestures, posture adjustments, or expressive movements. All behaviors were coded using predefined frequency thresholds to ensure consistency across observers and sessions. Observers completed joint calibration sessions prior to data collection to ensure shared interpretation and consistent scoring.

During VR exposure, one of the observers was positioned in close proximity to the athlete (approximately 1 m) to ensure immediate physical support if needed, particularly in cases where an athlete attempted to stand, move, or interact with the environment while wearing the headset. This positioning was selected to minimize the risk of falls or collisions during immersive exposure and to allow rapid intervention in the event of unexpected movements.

Monitoring for Cyber sickness and Discomfort: Using SSQ-aligned observational criteria, staff monitored early signs of VR-induced discomfort, including: eye rubbing, excessive blinking, or gaze aversion, postural instability, sudden stepping, or bracing actions, facial tension, pallor, or other indicators of nausea, behaviors suggestive of disorientation or sensory overload.

Athletes were permitted to stop the session at any time.

Usability and Technical Logging: All technical issues, including tracking loss, video orientation errors, accidental menu activation, battery depletion, overheating, and controller-related disruptions, were systematically logged.

User-related challenges, such as difficulty maintaining head alignment, interpreting prompts, or managing sensory flow, were recorded in parallel to support feasibility evaluation.

#### 2.3.3 Post-session assessments

Emotional State (Post-VR): The same emoji-based scale used during the pre-session emotional assessment was used to capture immediate emotional responses after headset removal.

Post-VR Balance Assessment: The same static and dynamic balance tasks as the pre-session were repeated to detect short-term VR-related changes.

A short, structured debrief was conducted using simple, concrete prompts supported by gestures and demonstrations to facilitate understanding. Prompts focused on immediately observable sensations and included questions such as: “Do you feel dizzy now?”, “Do your eyes feel tired?”, “Do you feel unsteady when standing?”, and “Do you want to stop or rest?”. Verbal prompts were accompanied by pointing to the head, eyes, or body, and by demonstrating standing still or taking a step, to support comprehension.

Session Repetition: The three VR sessions were conducted on separate training days with a consistent interval of six days between sessions for all participants. All sessions took place at the same time of day for each athlete to minimize potential effects of fatigue, circadian variation, or training schedule differences.

### 2.4 Data analysis

Descriptive statistics (means, standard deviations, frequencies, and percentages) were applied to summarize emotional ratings, behavioral indicators, balance scores, SSQ-aligned observations, and usability events across sessions. Due to the small sample size (*n* = 6) and the non-parametric nature of the measures, no inferential statistics were performed; all results are reported as group-level descriptive trends.

### 2.5 AI usage disclosure

Generative AI tools [ChatGPT version OpenAI GPT-5.1, Grammarly (v1.2.215.1793)] were used exclusively to support linguistic refinement and organization of the Methods text; all methodological descriptions, analyses, and interpretations were developed, verified, and approved by the authors.

## 3 Limitations

This study has several limitations. The small sample size (*n* = 6) limits generalizability and allows only descriptive analysis. Participants were all male athletes from a single adapted basketball program, reducing demographic and sport-specific diversity. The behavioral and balance scoring tools were feasibility-oriented adaptations rather than validated instruments, which may restrict their precision. The strong familiarity and trust between athletes and the research team may have positively influenced comfort and usability outcomes. Finally, the findings reflect responses to non-interactive, low-motion VR content and may not generalize to other VR formats or more complex interactive tasks.

## 4 Results

### 4.1 Emotional responses to VR exposure

Overall pattern favors mood stabilization or improvement across sessions.

Across sessions, the overall emotional profile remained positive, with most ratings classified as Good both before (77.8%) and after (77.8%) exposure (Table 2).

**Table 2:** Transition matrix: pre → post VR across all sessions.

Pre VR	Post: good	Post: neutral	Post: bad	Row total
Good	11 (61.1%)	0 (0%)	1 (5.6%)	12 (66.7%)
Neutral	3 (16.7%)	0 (0%)	0 (0%)	3 (16.7%)
Bad	2 (11.1%)	0 (0%)	1 (5.6%)	3 (16.7%)
Column total	16 (88.9%)	0 (0%)	2 (11.1%)	18 (100%)

Initial exposure (Session 1) produced a balanced mix of Good and Neutral responses, consistent with mild anticipatory tension before and adaptive curiosity after the session. One athlete reported a Bad mood. When asked to explain his response, he said it was because his favorite ball was missing at the basketball hall.

In Session 2, the same athlete reported a transient Bad mood after the session, explaining his choice with his favorite color - red. By the third VR exposure, all athletes reported feeling “Good” (100%) after, suggesting growing emotional familiarity and comfort with the virtual environment. The only bad mood response was not related to the VR session but linked to a color preference.

The transition matrix indicates that positive emotional states were generally stable, while negative or neutral reports tended to shift toward improvement rather than deterioration.

Collectively, these findings demonstrate that short-term VR exposure did not elicit sustained negative emotions or distress in athletes with ID. Instead, repeated sessions appeared to foster increasing positive affect and emotional adaptation, supporting the safe and tolerable nature of the VR-based training protocol.

### 4.2 Behavioral indicators during VR

Behavioral observations across the three VR training sessions demonstrated a consistent pattern of increased engagement and decreased behavioral signs associated with uncertainty, distraction, or discomfort. These indicators offer an objective perspective on how athletes with ID adapted to immersive VR.

Mean engagement levels increased progressively across sessions, from Session 1 (M = 1.67, SD = 1.21) to Session 2 (M = 2.17, SD = 0.98) and Session 3 (M = 2.83, SD = 0.41), with an overall mean of M = 2.22 (SD = 0.78). This progression reflected a shift from cautious initial participation to more sustained attention and involvement. In parallel, distraction episodes declined over time, showing high variability in Session 1 (M = 7.83, SD = 12.35), followed by lower values in Session 2 (M = 4.17, SD = 5.67) and Session 3 (M = 2.00, SD = 1.67). The overall mean for distraction episodes across sessions was M = 4.67 (SD = 6.55). This trend suggests improved sensory tolerance and greater attentional stability as VR became more familiar. The frequency of hesitation events was highest during Session 1 (M = 0.83, SD = 1.33), whereas substantially lower values were observed in Sessions 2 and 3 (M = 0.17, SD = 0.41 for both sessions). The overall mean across was M = 0.39 (SD = 0.68), indicating a progressive reduction in uncertainty over the course of exposure. Verbal expressions increased progressively across sessions, from Session 1 (M = 9.83, SD = 9.22) to Session 3 (M = 15.83, SD = 11.91), with an overall mean of M = 12.72 (SD = 10.46). This increase in verbalization reflects heightened engagement, curiosity, and affective expression as participants became more familiar and comfortable with the VR environment. Similarly, physical cues (e.g., spontaneous gestures or expressive movements) exhibited an upward trend, rising from Session 1 (M = 2.00, SD = 2.61) through Session 2 (M = 2.83, SD = 3.13) to Session 3 (M = 3.33, SD = 4.55), with an overall mean of M = 2.72 (SD = 3.01). Although modest, this progression suggests improved embodiment and reduced task-related tension.

### 4.3 Balance performance before and after VR exposure

Balance assessments conducted immediately before and after each VR exposure showed no evidence of postural deterioration. Instead, athletes demonstrated stable or improved performance across both static and dynamic tasks.

#### 4.3.1 Pre-VR baseline balance

Balance assessments conducted immediately before and after each VR, exposure revealed no evidence of postural deterioration following immersive VR viewing. Across sessions, athletes demonstrated either stable or improved performance in both static and dynamic balance tasks.

At baseline, athletes displayed heterogeneous balance profiles, consistent with typical inter-individual variability among persons with intellectual disabilities. Four athletes (Athletes 2, 3, 5, and 6) demonstrated relatively stable posture and coordinated gait, characterized by minimal sway and accurate step placement. In contrast, two athletes (Athletes 1 and 4) exhibited moderate instability, including observable postural sway, compensatory trunk movements, and deviations during heel-to-toe walking. Group-level mean raw balance scores reflected this variability, with mean summed scores of 4.0 for static balance (0–12 scale) and 4.7 for dynamic balance (0–15 scale).

Following VR exposure, no athlete demonstrated a decline in either static or dynamic balance performance. Instead, all participants either maintained or improved their balance scores Mean raw post-VR scores decreased to 1.3 for both static (0–12 scale) and dynamic (0–15 scale) balance, indicating greater postural stability (lower scores reflect better performance; Table 3).

**Table 3:** Pre - post balance scores across all athletes.

Athlete	Static pre	Static post	*Δ* static	Dynamic pre	Dynamic post	*Δ* dynamic
Athlete 1	8	4	– 4	8	4	– 4
Athlete 2	3	0	– 3	4	0	– 4
Athlete 3	0	0	0	0	0	0
Athlete 4	7	4	– 3	8	4	– 4
Athlete 5	3	0	– 3	4	0	– 4
Athlete 6	3	0	– 3	4	0	– 4

Values are summed raw observational scores, lower scores indicate better balance and *Δ* = Post - Pre (negative values indicate improvement).

Notably, four athletes achieved optimal post-VR scores (score = 0) on both balance tasks. Athletes who exhibited the weakest baseline stability (Athletes 1 and 4) showed improvements, with score reductions of up to four points.

Across all post-exposure assessments, no increases in sway, missteps, disorientation, or signs of vestibular-visual conflict were observed. No athlete required additional support, rest, or recovery time following VR exposure. Overall, these findings indicate that exposure to low-motion, non-interactive 360° VR content did not negatively affect motor stability and was physiologically well tolerated by athletes with intellectual disabilities.

### 4.4 SSQ-Aligned observational indicators

Throughout the VR exposures, SSQ-aligned observational monitoring indicated a high level of tolerability. Only infrequent and mild oculomotor or disorientation-related signs were observed in two athletes, all of which remained within the negligible severity range. No nausea-related indicators were recorded at any time. Taken together, these findings indicate that low-motion, non-interactive 360° VR exposure was well tolerated by athletes with intellectual disabilities, with no clinically relevant simulator sickness observed (Table 4).

**Table 4:** Summary of SSQ-aligned observational indicators across all athletes and sessions.

Measure	Value
Total number of athletes	6
Total VR sessions assessed	18
Total SSQ observations	324
Observations with any symptom (score > 0)	6 (1.9%)
Observations without symptoms (score = 0)	318 (98.1%)
Athletes exhibiting any symptoms	2 of 6
Mean number of symptoms per athlete	1.0
Nausea-related symptoms	None observed
Oculomotor-related symptoms	Mild, infrequent
Disorientation-related symptoms	Mild, infrequent
Overall SSQ severity classification	Negligible (<5)

### 4.5 Technical and user-related challenges

A structured usability log was maintained during all VR sessions to document device-related, cognitive, and procedural challenges that could affect comfort, safety, or continuity of use. Although issues were generally minor, they provided necessary guidance for implementing VR in adapted sport settings for athletes with ID.

#### 4.5.1 Headset comfort and visual calibration

All athletes tolerated the Meta Quest 3 well. No adjustments were needed during sessions, and no discomfort related to strap tension, headset weight, or eye alignment was observed. These findings indicate that the device's ergonomic design was appropriate for this population.

No athlete showed signs of blurred vision or IPD misalignment, and recalibration was never required. The main visual challenge involved 360° video orientation: when videos started facing a different direction than the athlete, assistance was needed to orient to the correct viewpoint. Video initiation also proved sensitive to headset transfer, often causing pauses or resets. Because most athletes could not restart content by themselves, researchers intervened frequently. Continuous casting enabled rapid detection and correction of these disruptions.

#### 4.5.2 Controller interaction and cognitive demands

Controller use was a significant usability barrier. Only two athletes demonstrated purposeful controller interaction; others triggered unintended functions through hand movements, including accidental menu activation and video interruption. In some cases, sessions needed to be restarted. When used correctly, pointing and trigger activation were performed without difficulty, though this was limited to a small subset of participants.

Casting the VR view to an external monitor functioned reliably, with only brief lags that did not affect safety or session flow. Instructors maintained continuous visual access to the athlete's perspective, supporting timely troubleshooting.

Several athletes experienced difficulty interpreting on-screen prompts and required consistent verbal scaffolding. Despite this, transitions between virtual and real-world tasks remained smooth, and no confusion emerged during headset removal or repositioning.

No athlete demonstrated resistance to the headset, heightened arousal, or behavioral withdrawal. Sessions proceeded without the need for breaks or emotional redirection, indicating high acceptance of VR.

The session environment supported stable tracking and presented minimal distractions. No modifications to lighting, space, or layout were required.

#### 4.5.3 Session management and operational barriers

Logistical challenges included extended setup time and highly challenging cleaning and disinfection, occasional technical interruptions, rapid battery depletion of both headset and controllers, and intermittent device overheating. These factors increased staff workload and should be anticipated when planning VR use with multiple athletes.

## 5 Discussion

This study evaluated the feasibility, usability, and individual experience of immersive virtual reality (VR) in athletes with intellectual disabilities (ID) participating in adapted basketball training. By synthesizing observations of emotional, behavioral, physiological, and usability factors, the findings show that short, structured VR exposure was well tolerated and operationally manageable, supporting its preliminary feasibility in adapted sports environments.

Across all three sessions, athletes demonstrated consistently positive emotional responses. Good mood remained stable before and after VR exposure, and no negative effect appeared in the final session. This pattern aligns with prior work indicating that individuals with ID typically respond well to visually structured and predictable VR environments when cognitive demands are minimized (2, 9). Notably, the few early negative ratings stemmed from non-VR contextual factors, reinforcing evidence that low-motion, non-interactive VR can maintain psychological safety in vulnerable users (1, 3).

Behavioral indicators showed clear adaptive progression: engagement increased substantially, while hesitation and distraction declined across sessions. These trends suggest improved attentional stabilization and familiarity with immersive visual flow - consistent with studies demonstrating that repeated VR experience reduces cognitive ambiguity and enhances focus in people with ID (6, 8). Only one athlete discontinued the first session early, and no participant exhibited avoidance, agitation, or sensory distress, behaviors sometimes observed among individuals with anxiety or sensory hypersensitivity. This supports the suitability of gradual familiarization and the use of stable, non-interactive 360° sport content during initial VR use.

Usability findings further support feasibility while highlighting practical constraints. The adjustment and fitting process was notably easy for the research team because the athletes were familiar with them and demonstrated a high level of trust, which likely reduced initial anxiety and facilitated smooth setup. The headset was well tolerated, and no athlete experienced discomfort or sensory intolerance - consistent with research suggesting that modern lightweight VR devices are generally acceptable to users with mild and moderate ID (10). In contrast, controller operation proved challenging for most athletes. Only two participants used controllers purposefully, while others inadvertently triggered menus or paused videos, aligning with literature documenting visuomotor and symbolic-processing challenges among VR users with ID (12, 13). These results indicate that non-interactive VR is currently the most accessible modality for athletes with ID in early-stage adapted sports training.

Technical issues such as video-start interruptions, rapid battery depletion, minor tracking loss, and occasional overheating were present but manageable. These challenges are consistent with reports from applied VR studies and underscore the need for efficient session management (2, 12, 13). Real-time casting to an external display proved essential for monitoring orientation, troubleshooting disruptions, and maintaining safety - supporting existing recommendations for continuous supervision when using VR with populations with cognitive vulnerabilities (3).

Physiologically, the VR sessions were well tolerated. Pre-post balance scores remained stable or improved, contrasting with findings in neurotypical adults, who reported temporary postural disruption after immersive VR (11, 18, 19). The absence of sway increases or compensatory movements likely reflects the stable optical flow and low motion of the selected content (5, 11). Likewise, SSQ-aligned observational scoring revealed insignificant symptoms in nearly all entries, with no nausea recorded, an unusually low cyber sickness profile for VR research.

This dataset provides real-world evidence on how athletes with ID respond to immersive VR within an active, sports-based context - a setting largely absent in existing VR/ID research. Unlike most prior studies focusing on educational or therapeutic applications, this study examines how VR use could be applied within adapted basketball training, combining emotional, behavioral, and motor outcomes with detailed usability mapping.

Overall, the findings indicate that immersive VR, when delivered through stable, low-motion content and supported by continuous supervision, can be feasibly and safely integrated into adapted basketball training for athletes with ID. Further research with larger, more diverse samples is needed to refine protocols, examine long-term effects, and explore the potential for progressively interactive VR modalities.

## Data Availability

The raw data supporting the conclusions of this article will be made available by the authors, without undue reservation.
